# Evaluation of the Cytotoxicity and Genotoxicity of Flavonolignans in Different Cellular Models

**DOI:** 10.3390/nu9121356

**Published:** 2017-12-14

**Authors:** Michal Bijak, Ewelina Synowiec, Przemyslaw Sitarek, Tomasz Sliwiński, Joanna Saluk-Bijak

**Affiliations:** 1 Department of General Biochemistry, Faculty of Biology and Environmental Protection, University of Lodz, Pomorska 141/143, 90-236 Lodz, Poland; joanna.saluk@biol.uni.lodz.pl; 2 Laboratory of Medical Genetics, Faculty of Biology and Environmental Protection, University of Lodz, Pomorska 141/143, 90-236 Lodz, Poland; ewelina.synowiec@biol.uni.lodz.pl (E.S.); tomasz.sliwinski@biol.uni.lodz.pl (T.S.); 3 Department of Biology and Pharmaceutical Botany, Medical University of Lodz, Muszynskiego 1, 90-151 Lodz, Poland; przemyslaw.sitarek@umed.lodz.pl

**Keywords:** flavonolignans, silybin, silychristin, silydianin, blood platelets, mitochondria, ROS, cytotoxicity, genotoxicity

## Abstract

Flavonolignans are the main components of silymarin, which represents 1.5–3% of the dry fruit weight of Milk thistle (*Silybum marianum* L. Gaernt.). In ancient Greece and Romania, physicians and herbalists used the *Silybum marianum* to treat a range of liver diseases. Besides their hepatoprotective action, silymarin flavonolignans have many other healthy properties, such as anti-platelet and anti-inflammatory actions. The aim of this study was to evaluate the toxic effect of flavonolignans on blood platelets, peripheral blood mononuclear cells (PBMCs) and human lung cancer cell line—A549—using different molecular techniques. We established that three major flavonolignans: silybin, silychristin and silydianin, in concentrations of up to 100 µM, have neither a cytotoxic nor genotoxic effect on blood platelets, PMBCs and A549. We also saw that silybin and silychristin have a protective effect on cellular mitochondria, observed as a reduction of spontaneous mitochondrial DNA (mtDNA) damage in A549, measured as mtDNA copies, and mtDNA lesions in *ND1* and *ND5* genes. Additionally, we observed that flavonolignans increase the blood platelets’ mitochondrial membrane potential and reduce the generation of reactive oxygen species in blood platelets. Our current findings show for the first time that the three major flavonolignans, silybin, silychristin and silydianin, do not have any cytotoxicity and genotoxicity in various cellular models, and that they actually protect cellular mitochondria. This proves that the antiplatelet and anti-inflammatory effect of these compounds is part of our molecular health mechanisms.

## 1. Introduction

Milk thistle (*Silybum marianum* L. Gaernt.), sometimes called “wild artichoke”, is a medicinal plant that has been used for thousands of years as a remedy for a variety of ailments [1]. There is a lot of epidemiological evidences that plant secondary metabolites have various health activities—protected against development of cancers, cardiovascular diseases, diabetes, osteoporosis as well as neurodegenerative diseases [2]. In ancient Greece and Romania, physicians and herbalists used *Silybum marianum* to treat a range of liver and gallbladder disorders, including hepatitis, cirrhosis and jaundice, as well as to protect the liver against poisoning from chemical and environmental toxins [3]. By the 19th century, American Eclectic physicians used milk thistle for varicose veins and liver, spleen and kidney disorders, while in the mid-19th century, the German physician Rademacher used the milk thistle fruit for treatment of liver diseases [4]. Needless to say, its hepatoprotective action has been proven by many studies [5,6,7,8,9,10].

Silymarin represents 1.5–3% of the fruit’s dry weight and is an isomeric mixture of flavonoid complexes—flavonolignans. These compounds possess a unique chemical structure, which is composed of two main units. The first is based on a taxifolin, which is a flavanonol group in flavonoids. The second is a phenylpropanoid unit [11,12]. The main representatives of this group that are present in silymarin are silybin, isosilybin, silychristin, isosilychristin silydianin and silymonin [3,13,14,15,16,17]. The main silymarin flavonolignans are silybin, silychristin and silydianin, which form approximately 90% of the extract’s composition. The silybin content is approximately 50% to 70%; silychristin, about 20%, and silydianin, about 10% of silymarin composition [3].

Besides their hepatoprotective action, silymarin flavonolignans have many other healthy properties [18]. It has been established that these compounds have strong antioxidative properties and modulate a variety cell-signaling pathways, resulting in the reduction of pro-inflammatory mediators [19]. There is a strong body of evidence that silymarin is also a potential anticancer and chemopreventive agent [20,21,22,23,24,25].

Research performed by our team demonstrated that three major silymarin flavonolignans—silybin, silychristin and silydianin—are able to reduce blood platelet activation in different physiological pathways. These compounds were shown to reduce ADP-induced blood platelet activation and aggregation in whole blood samples [26], as well as collagen-induced blood platelets’ activation, adhesion, aggregation and secretion of Platelet Factor 4 (PF-4) [27]. We also determined the effect of these flavonolignans on arachidonic acid pathway blood platelets, and showed that the compounds that we tested decrease platelet aggregation level—both thromboxane A2 and malondialdehyde formation, as well as inhibiting cyclooxygenase activity [28]. The other positive effect observed in our previous study was a reduction in cross-talk between hemostasis and the immunological system. We observed that silybin and silychristin inhibit the IL-1β-induced formation of blood platelet-leukocyte aggregates in whole blood samples in a dose-dependent manner, as well as leucocyte production of the pro-inflammatory cytokines-IL-2, TNF, INF-α, and INF-γ. Additionally, these two flavonolignans abolished the IL-1β-induced expression of mRNA for IFN-γ and TNF in leucocytes [29].

Based on the results obtained in our previous studies, and to confirm that all the observed positive effects are related to healthy mechanisms of the action of flavonolignans, we aimed to evaluate the toxic effect of silybin, silychristin and silydianin on blood platelets, peripheral blood mononuclear cell (PBMC) and human lung cancer cell line—A549—using different molecular techniques.

## 2. Materials and Methods

### 2.1. Reagents

Dimethyl sulfoxide (DMSO), 3-[(3-Cholamidopropyl)dimethylammonio]-1-propanesulfonate (CHAPS), 4-(2-Hydroxyethyl)piperazine-1-ethanesulfonic acid (HEPES), glucose, Tris, NADH, sodium pyruvate, Histopaque^®^-1077, RPMI 1640 and DMEM mediums as well as the flavonolignans (silybin, silychristin and silydianin), were all obtained from the Sigma-Aldrich Chemical Co. (St. Louis, MO, USA). Trypan blue dye and cell counter slides were sourced from Bio-Rad (Hercules, California, USA). Both the JC-1 Dye and 2′,7′-dichlorodihydrofluorescein diacetate (DCFH-DA Dye), came from Thermo Fisher Scientific (Waltham, MA, USA). All other chemicals were reagent grade or the highest-quality available.

### 2.2. Blood Samples

Blood samples collected from 12 different healthy donors were purchased from the Regional Centre for Transfusion Medicine in Lodz (Lodz, Poland). All samples were drawn in the morning, from fasting donors. All donors were checked by a doctor and found to have no cardiovascular disorders, allergies, lipid or carbohydrate metabolism disorders, nor were they being treated with any drugs [30]. Our analysis of the blood samples was performed under the guidelines of the Helsinki Declaration for Human Research, and approved by the Committee on the Ethics of Research in Human Experimentation at the University of Lodz (Resolution No. 16/KBBN-UŁ/II/2016).

### 2.3. Blood Platelets’ Isolation and Sample Preparation

The blood was centrifuged (200× *g*, 10 min, room temperature—RT) to isolate the platelet rich plasma (PRP). Blood platelets were isolated from PRP using BSA–Sepharose 2B gel filtration, according to Walkowiak [31]. After isolation, the platelets were suspended in a modified Tyrode’s Ca^2+^/Mg^2+^ free buffer (127 mM NaCl, 2.7 mM KCl, 0.5 mM NaH_2_PO_4_, 12 mM NaHCO_3_, 5 mM HEPES, 5.6 mM glucose, pH 7.4). Platelet suspensions (2 × 10^8^ platelets/mL) were pre-incubated with the silybin, silychristin and silydianin in 3 concentrations (10–50–100 µM) for 30 min at 37 °C. All tested compounds were initially dissolved in 20% DMSO to a preliminary concentration of 20 mM. Other solutions of the compounds used were also performed in 20% DMSO (prepared in 50 mM TBS, pH 7.4). The final DMSO concentration of all samples was 0.1%. In control samples the same volume of solvent was added (20% DMSO prepared in 50 mM TBS, pH 7.4), with the probes warmed for 30 min at 37 °C.

### 2.4. Blood Platelets’ Mitochondrial Membrane Potential (MMP)

To measure MMP the fluorescent dye JC-1 (5′,6,6′-tetrachloro-1,1′,3,3′-tetraethylbenzimidazolylcarbocyanine iodide) was used [32]. The ratio of red to green fluorescence of JC-1 is dependent only on membrane potential, and is not influenced by mitochondrial size, shape, or density. Blood platelet samples were pre-incubated with 5 μM JC-1 (prepared in Tyrode’s Ca^2+^/Mg^2+^ free buffer), at 37 °C for 30 min. The fluorescence was measured on a Bio-Tek Synergy HT Microplate Reader (Bio-Tek Instruments, Winooski, VT, USA), with filter pairs of 530 nm/590 nm and 485 nm/538 nm. Results are shown herein as a ratio of fluorescence, measured at 530 nm/590 nm to that measured at 485 nm/538 nm (aggregates to monomer fluorescence).

### 2.5. Measurement of Intracellular Reactive Oxygen Species (ROS) Levels

The relative level of intracellular ROS in blood platelets was measured using the redox-sensitive fluorescent dye-DCFH-DA [33]. Blood platelet samples were pre-incubated with 5 μM of DCFH-DA (prepared in Tyrode’s Ca^2+^/Mg^2+^ free buffer), at 37 °C for 45 min. Fluorescence was measured at a 480 nm excitation wavelength and an emission wavelength of 510 nm, using a Bio-Tek Synergy HT Microplate Reader (Bio-Tek Instruments, Winooski, VT, USA), and expressed as a percentage of the control (blood platelets without tested compounds).

### 2.6. Generation of Superoxide Anion Radicals (O_2_⁻•)

Generation of superoxide anion radicals (O_2_⁻•) in blood platelets was measured using the cytochrome C reduction method, as described earlier [34,35]. For that, 250 µL of cytochrome C (160 µM) prepared in Ca^2+^/Mg^2+^ free Tyrode’s buffer was added to an equal volume of platelet suspensions in the same buffer. After incubation (30 min at 37 °C), the platelets were sedimented by centrifugation at 2000× *g* for 5 min, and the supernatants (200 µL) were added to the microplatelet wells. Reduction of cytochrome C was measured spectrophotometrically at 550 nm. To calculate the molar concentration of O_2_⁻•, an extinction coefficient for cytochrome C of 18,700 M^−1^∙cm^−1^ was used.

### 2.7. Lactic Dehydrogenase Releasing from Blood Platelets

Blood platelet viability and the cytotoxic effects of tested flavonolignans were determined by measuring the activity of lactic dehydrogenase—LDH (cell lysis marker)—in an extracellular medium (PPP) using the spectrophotometric method described by Wróblewski and LaDue [36]. The PRP samples were pre-incubated with the silybin, silychristin and silydianin in 3 concentrations (10–50–100 µM) for 30 min at 37 °C. All tested compounds were initially dissolved in 20% DMSO (prepared in 50 mM TBS, pH 7.4), to a preliminary concentration of 20 mM. After that, the samples were centrifuged (1000× *g*, 10 min, RT). Into the microplate (Corning^®^ Brand 96-Well UV Plates) reaction wells, 270 µL of 0.1 M phosphate buffer (pH 7.4), 10 µL of the obtained PPP, and 10 µL of NADH were then all added. After 20 min, 10 µL of sodium pyruvate was added to all reaction wells using a multichannel pipette, thus all the reactions began simultaneously. The absorbance measurements were performed in a 96-well microplate reader (SPECTROstarNano, BMG Labtech, Ortenberg, Germany), at ambient temperature. The absorbance values were monitored every 60 s for 10 min. Lactic dehydrogenase activity was determined by a decrease of absorbance at 340 nm, and expressed as a percentage of the control (PPP obtained from PRP without tested compounds).

### 2.8. Peripheral Blood Mononuclear Cell Viability Assay

A PBMC fraction was isolated from human blood, obtained and collected as described above, with a density gradient centrifugation method using a Histopaque^®^-1077 (a sterile solution of polysucrose, 57 g/L, and sodium diatrizoate, 90 g/L, with a density of 1.077 g/mL). The blood was carefully layered (in a volume ratio of 1:1) onto the Histopaque^®^-1077, and centrifuged for 30 min (400× *g*, at room temperature). Afterwards, the collected pellet was washed twice with RPMI 1640 (400× *g*, 10 min at room temperature). The obtained fraction of PBMCs was suspended in RPMI 1640 to suspension 1 × 10^6^ PBMCs/mL. The PBMC samples were pre-incubated with the flavonolignans (silybin, silychristin and silydianin), in 3 concentrations (10–50–100 µM) for 1 h at 37 °C. All tested compounds were initially dissolved in 20% DMSO (prepared in 50 mM TBS, pH 7.4) to the preliminary concentration of 20 mM. Cell viability (%) was determined during a spectrofluorimetric analysis, involving the use of propidium iodide as a fluorescent dye. Measurements were conducted using a microchip-type automatic cell counter Adam-MC DigitalBio (NanoEnTek Inc., Seoul, Korea) according to the manufacturer’s protocol.

### 2.9. Cell Cultures

The cytotoxicity and genotoxicity experiments were performed on a human lung cancer cell line—A549 (CCL-185; ATCC) cell line, obtained from American Type Culture Collection (ATCC™, Manassas, VA, USA). The cell line was placed in a humidified incubator with a 5% CO_2_ atmosphere at 37 °C in a DMEM medium, supplemented with 10% (*v*/*v*) heat-inactivated Fetal Bovine Serum (FBS), 100 U/mL penicillin, and 100 μg/mL streptomycin. Cell culture reagents were obtained from Lonza (Basel, Switzerland). For cytotoxicity and genotoxicity, cells were seeded at 3 × 10^6^ cells per well and were left overnight before treatment. The following day, the cell samples were incubated with flavonolignans (silybin, silychristin and silydianin), in 3 concentrations (10–50–100 µM) for 24 h.

### 2.10. Cell Viability Determination

The viability of A549 cells treated with different concentrations of flavonolignans was estimated using a Bio-Rad TC20 automated cell counter (Hercules, CA, USA), using trypan blue dye (Bio-Rad Laboratories, Hercules, CA, USA) according to the manufacturer’s protocol. Cell viability was expressed as a percentage relative to the untreated (control) cells, defined as 100%.

### 2.11. DNA Extraction from Cell Cultures

Treated cell suspensions were collected by centrifugation (400× *g*, 12 min) and total genomic DNA (nuclear and mitochondrial) was isolated using the QIAamp DNA Mini Kit (Qiagen, Valencia, CA, USA) according to the manufacturer’s instructions. The DNA concentrations were determined by spectrophotometric measurement of absorbance at 260 nm, and the purities were calculated at a ratio of A260/A280 using a Bio-Tek Synergy HT Microplate Reader (Bio-Tek Instruments, Winooski, VT, USA). The purified DNA was stored at −32 °C until further analysis.

### 2.12. Mitochondrial DNA Copy Number Quantification

The relative number of copies of human mitochondrial DNA using nuclear DNA (nDNA) content as a standard was assessed by quantitative real-time PCR (qRT-PCR). To this quantification two primer pairs for detecting mtDNA (*ND1*, *ND5*), and two primer pairs for detecting nDNA (SLCO2B1, SERPINA1), were selected. All primers were designed with the help of the Primer3 software (http://bioinfo.ut.ee/primer3-0.4.0/) and synthesized by Sigma-Aldrich (St. Louis, MO, USA). Complete nucleotide sequences for each gene were taken from the ENSEMBL database (https://ensembl.org/). Mitochondrial *ND1* (124 bp fragment size), and *ND5* genes (124 bp fragment size), were amplified using the primer pair (forward primer 5′-CCTAAAACCCGCCACATCTA-3′ and reverse primer 5′-GCCTAGGTTGAGGTTGACCA-3′; forward primer 5′-AGGCGCTATCACCACTCTGT-3′ and reverse primer 5′-TTGGTTGATGCCGATTGTAA-3′), respectively. For determination of the amount of nuclear DNA, the SLCO2B1 (135 bp fragment size) and SERPINA1 (148 bp fragment size) genes were used as a reference: forward primer 5′-TGCAGCTTCCTCTTCACAGA-3′ and reverse primer5′-CTCAGCCCCAAGTATCTCCA-3′; forward primer 5′-GATCCCAGCCAGTGGACTTA-3′ and reverse primer: 5′-CCTGAAGCTGAGGAGACAGG-3′.

qRT-PCR was amplified using a CFX96 real-time PCR system (Bio-Rad Laboratories, Hercules, CA, USA). A reaction mix of 10 µL contained 1 × RT PCR Mix SYBR A (A&A Biotechnology, Gdynia, Poland), 250 nM of each primer, and 1 μL (5 ng) of isolated genomic DNA. The RT-PCR conditions were as follows: 95 °С for 3 min, followed by 40 cycles at 95 °С for 15 s, 65 °С for 30 s, and 72 °С for 15 s, with plate reading at this step. Each reaction was performed in duplicate and included a negative control (without template DNA). The cycle threshold (Ct) values were calculated automatically and analyzed using the CFX Manager^TM^ Software (version 3.1). The relative mtDNA copy number was calculated using the formula 2^ΔCt1 and ΔCt2^, where ΔCt1 = Ct for *SLCO2B1*−Ct for *ND1*; ΔCt2 = Ct for *SERPINA1*−Ct for *ND5*.

### 2.13. Determination of Mitochondrial and Nuclear DNA Damage—Semi-Long Run qRT-PCR (SLR-qRT-PCR)

To assess the mitochondrial DNA (mtDNA) and nuclear DNA (nDNA) damage, the semi-long run quantitative RT-PCR (SLR-qRT-PCR) was used [37]. To measure the levels of the DNA lesions in the tested region of the mitochondrial or nuclear genome, two fragments of different lengths were used, i.e., long and small fragments, located in the same mitochondrial/nuclear genomic region. The sequence of all primers used in this study are listed in Table S1 in supplementary material.

SLR-qRT-PCR amplification was performed using a CFX96 real-time PCR system (Bio-Rad Laboratories, Hercules, CA, USA). The SLR-qRT-PCR reaction mix of 10 µL consisted of 1 × RT PCR Mix SYBR A (A&A Biotechnology, Gdynia, Poland), 250 nM of each primer and 1 ng of template DNA. The cycling sequence was as follows: initial denaturation of 3 min at 95 °C followed by up to 40 cycles of 15 s at 95 °C, 30 s at 65 °C, and 15 s at 72 °C (for short amplicons), or 45 s at 72 °C (for long amplicons). The cycle threshold (Ct) values were calculated automatically and the analysis made using CFX Manager^TM^ Software, version 3.1 (Bio-Rad Laboratories, Hercules, CA, USA). DNA damage was calculated as lesion per 10 kb DNA of each region, by including the size of the respective long fragment: Lesion per 10 kb DNA = (1 − 2^−(Δlong − Δshort)^) × 10,000 (bp)/size of long fragment (bp), where ∆long and ∆short indicate differences in Ct value between non-treated control and treated samples. DNA isolated from the non-treated cells (controls) was used as the reference, whereas the Ct of the large and small mitochondrial/nuclear fragments was used for DNA damage quantification.

### 2.14. Isolation of RNA and Reverse Transcription to cDNA

Treated cell suspensions were collected by centrifugation (400× *g*, 12 min). RNAs were then extracted from pelleted cells using a GenElute™ Mammalian Total RNA Miniprep Kit Q-PCR (Sigma-Aldrich, St. Louis, MO, USA), following the manufacturer’s instructions. RNA concentrations were determined by spectrophotometric measurement of absorbance at 260 nm, and purity was calculated at a ratio of A260/A280 with a Bio-Tek Synergy HT Microplate Reader (BioTek Instruments, Inc., Winooski, VT, USA).

Total RNA (1 µg) was reverse transcribed into cDNA with a High-Capacity cDNA Reverse Transcription Kit (Applied Biosystems™, Waltham, MA, USA). All steps were performed according to the manufacturer’s recommendations.

### 2.15. Analysis of Apoptotic Gene Expression Level

Expression levels of apoptotic genes were obtained using the following TaqMan probes: Hs00608023_m1 for the *BCL2* gene; Hs00180269_m1 for the *BAX (BCL2 associated X)* gene; Hs00559441_m1 for the *APAF1* (*apoptotic peptidase activating factor 1*) gene; Hs00234387_m1 for the *CASP3* (*caspase 3*) gene; Hs01018151_m1 for the *CASP8* (*caspase 8*) gene; Hs00962278_m1 for the *CASP9* (*caspase 9*) gene, and Hs99999901_s1 as an endogenous control (the human *18S rRNA* gene, sourced from Life Technologies, Carlsbad, CA, USA). Real-time PCR analyses were performed using a CFX96 real-time PCR system (Bio-Rad Laboratories, Hercules, CA, USA), with a TaqMan Universal Master Mix II without UNG (Life Technologies, Carlsbad, CA, USA). All procedures were performed according to the manufacturers’ protocols. The *C*_t_ values were calculated automatically and the analyses performed using CFX Manager^TM^ Software (version 3.1). Relative expressions of the studied genes were calculated using the equation 2^−ΔCt^, where ΔCt = Ct target gene − Ct 18S rRNA.

### 2.16. Data Analysis

The statistical analysis was performed using StatsDirect statistical software V. 2.7.2 (Cheshire, UK). All experimental values presented in this study were expressed as mean ± standard deviation (SD). To analyze the normality of the distribution of results, the Shapiro-Wilk test was used. Next, the results were analyzed for equality of variance using Levene’s test. The significance of the differences between the values was analyzed using ANOVA, followed by Tukey’s range test for multiple comparisons (for data with normal distribution and equality of variance), and the Kruskal-Wallis test; *p* < 0.05 was accepted as statistically significant [38,39,40,41].

## 3. Results

### 3.1. Effect of Flavonolignans on Platelets Viability and MMP

**Figure 1:**
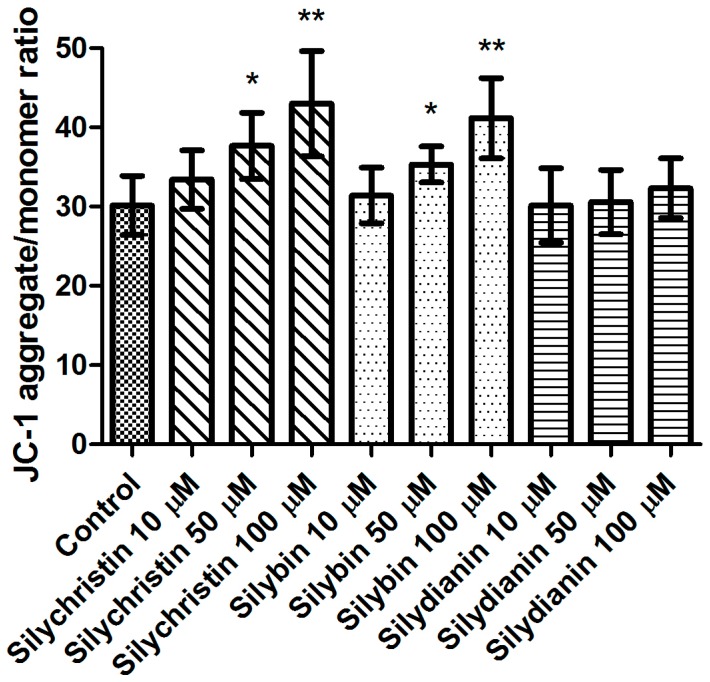
The effect of flavonolignans (silychristin, silybin and silydianin in concentrations of 10, 50 and 100 µM) on blood platelet mitochondrial membrane potential. MMP is expressed as a ratio of 530 nm/590 nm to 485 nm/538 nm (aggregates to monomer) fluorescence, as quantified with a fluorescent plate reader after JC-1 staining. The data represent means of ± standard deviation (SD), *n* = 12, * *p* < 0.05, ** *p* < 0.001.

**Figure 2:**
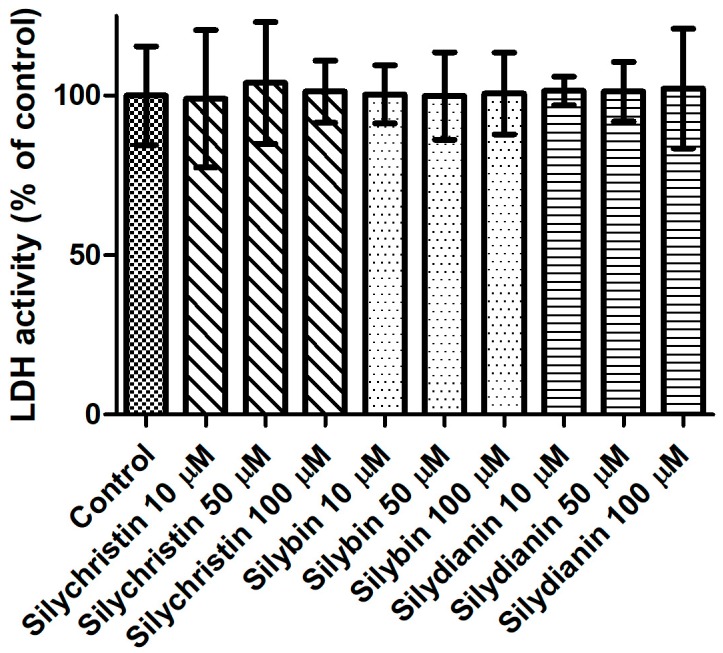
The effect of flavonolignans (silychristin, silybin and silydianin in concentrations of 10, 50 and 100 µM) on lactic dehydrogenase release from blood platelets. The results are expressed as a percentage of LDH activity in the control samples (without tested compounds). The data represent means of ± SD, *n* = 12.

In the first step of our study, we determined the effect of flavonolignans on the viability of blood platelets. At first, we checked the effect of flavonolignans on blood platelets’ mitochondrial membrane potential. We observed that the tested compounds did not reduce mitochondrial membrane potential in blood platelets (Figure 1). Moreover, in our experiments silychristin and silybin in 50 and 100 µM concentrations (*p* < 0.05 and *p* < 0.001 respectively), were seen to increase the blood platelets mitochondrial membrane potential. The cytotoxicity of flavonolignans on human blood platelets was also evaluated using estimation of LDH activity. None of the flavonolignans we used—silychristin, silybin and silydianin—in any of the tested concentrations (10, 50 and 100 µM) caused damage to blood platelets, as determined by lactate dehydrogenase activity in PPP (Figure 2).

### 3.2. Effect of Flavonolignans on ROS Generation in Platelets

**Figure 3:**
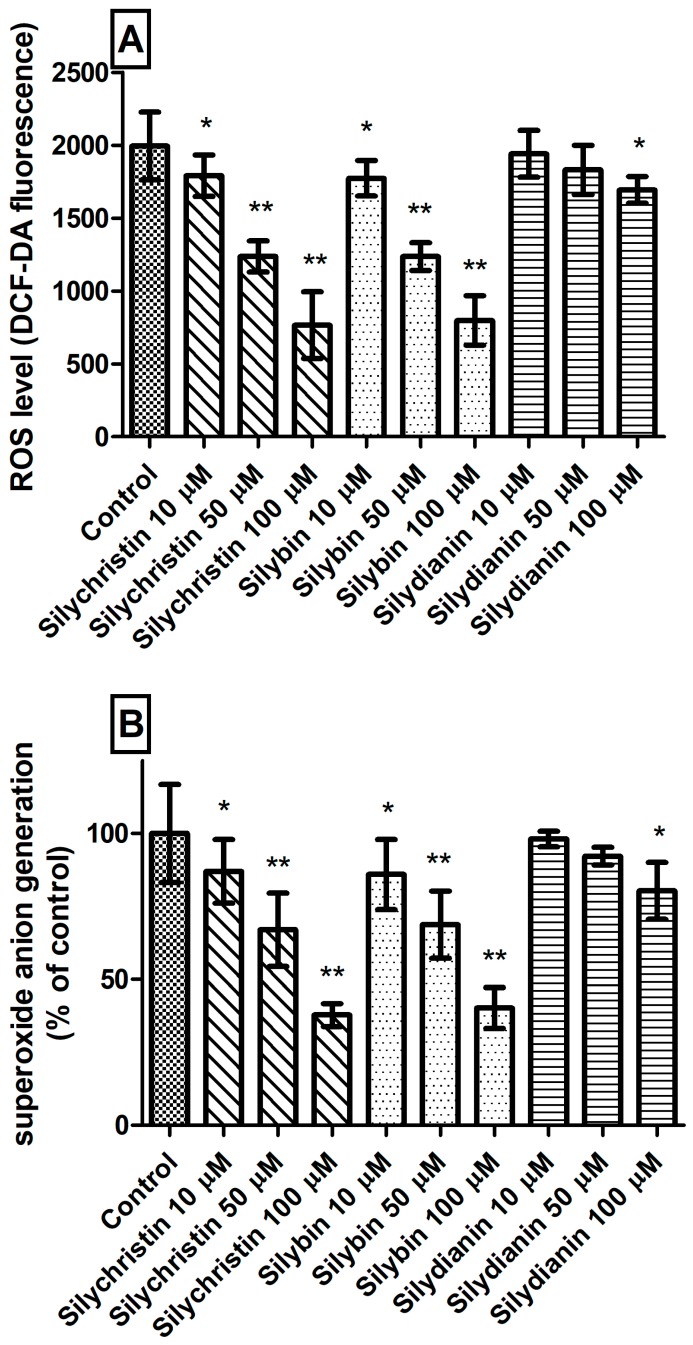
The effect of flavonolignans (silychristin, silybin and silydianin in concentrations of 10, 50 and 100 µM) on generation of reactive oxygen species in blood platelets. (**A**) intracellular ROS production measured as intensity of DCF fluorescence; (**B**) superoxide anion generation measured as cytochrome C reduction and expressed as a percentage value of the control sample. The data represent means of ± SD, *n* = 12. Statistical analysis was performed using Tukey’s Range Test, * *p* < 0.05, ** *p* < 0.001.

The next step in our studies of blood platelets was to measure ROS level generation. We observed that incubation with silychristin and silybin, in all tested concentrations, statistically significantly reduced intracellular ROS levels (Figure 3A) and generation of O_2_⁻• in blood platelets (Figure 3B). In the case of samples treated with silydianin, this effect was observed only for the highest tested concentration (100 µM).

### 3.3. Effect of Flavonolignans on PMBCs Viability

**Figure 4:**
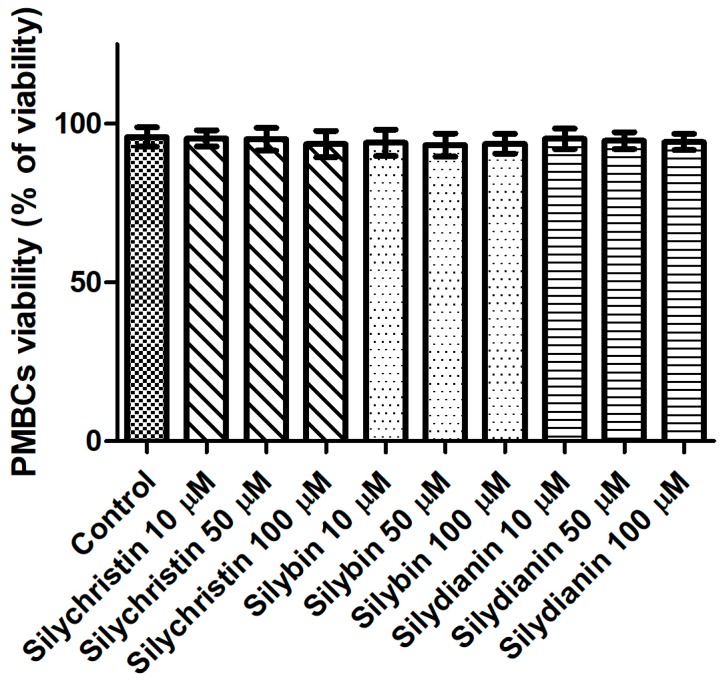
The effect of flavonolignans (silychristin, silybin and silydianin in concentrations of 10, 50 and 100 µM) on PMBCs viability. The data represent the mean of 8 measurements.

Next, we evaluated the potential cytotoxicity of tested flavonolignans using a model of PBMCs after 60 min of incubation. The lack of a toxic effect of flavonolignans was evidenced by a lack of significant differences (*p* > 0.05) in cell viability, observed between PBMCs incubated with flavonolignans (93.3–95.4% viability), and the control (untreated) samples (95.8 viability), regardless of the flavonolignans type and concentration (Figure 4).

### 3.4. Effect of Flavonolignans on Human Lung Cancer Cell Line A549 Viability

**Figure 5:**
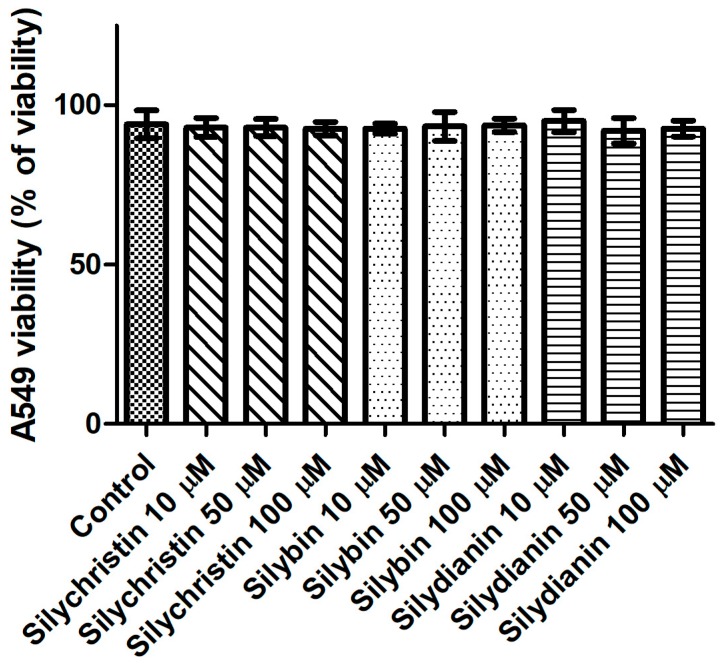
The effect of flavonolignans (silychristin, silybin and silydianin in concentrations of 10, 50 and 100 µM) on an A549 cell line, determined by trypan blue assay. The data represent the mean of 8 measurements.

To determine whether flavonolignans can affect cells, an assay was performed for in vitro cytotoxic activities in the standardized cell-culture system. For this assay, the human lung cancer cell line A549 was used. Cell morphology and cell viability were selected as the parameters for determining cytotoxicity. Cells were exposed to flavonolignans for 24 h, in different concentrations from 10 to 100 µM in the examined samples. For cell viability, Trypan blue exclusion assay was used. The result of testing flavonolignans on A549 human lung cancer cell line did not show any effects of cytotoxicity (*p* > 0.05), as shown in Figure 5. No agglutination, vacuolization, separation from the medium or cell membrane lyses were observed. In cultures which had contact with the investigated material samples, single rounded cells were noted at each observation time (Figure S1). After contact with these compounds, none of the cell cultures showed any damage, and the cells had proper morphologies and showed good proliferation in comparison to the control cells. Furthermore, proliferation of cells in the control and test cultures was similar, and the cells formed colonies across the entire surface of the plates.

### 3.5. Effect of Flavonolignans on mtDNA Copy Number in Human Lung Cancer Cell Line A549

**Figure 6:**
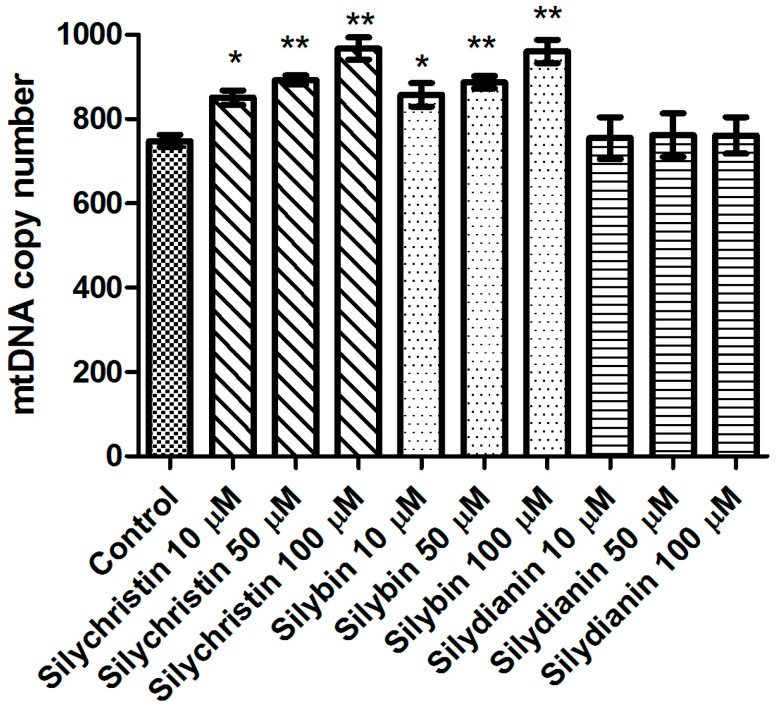
The effect of flavonolignans (silychristin, silybin and silydianin in concentrations of 10, 50 and 100 µM) on mitochondrial DNA copy number in an A549 cell line measured by real-time quantitative PCR. The data represent means of ± SD, *n* = 8. Statistical analysis was performed using the Kruskall-Wallis Test, * *p* < 0.05, ** *p* < 0.001.

Additionally, in this study we demonstrated that after 24 h of incubation with silydianin, the mtDNA copy number in the A549 cells is at the same level as in the untreated cells (Figure 6). After 24 h of treatment with silychristin and silybin in all tested concentrations, the cells had a higher (*p* < 0.05) mtDNA copy number.

### 3.6. Effect of Flavonolignans on mtDNA and nDNA Damages in Human Lung Cancer Cell Line A549

**Figure 7:**
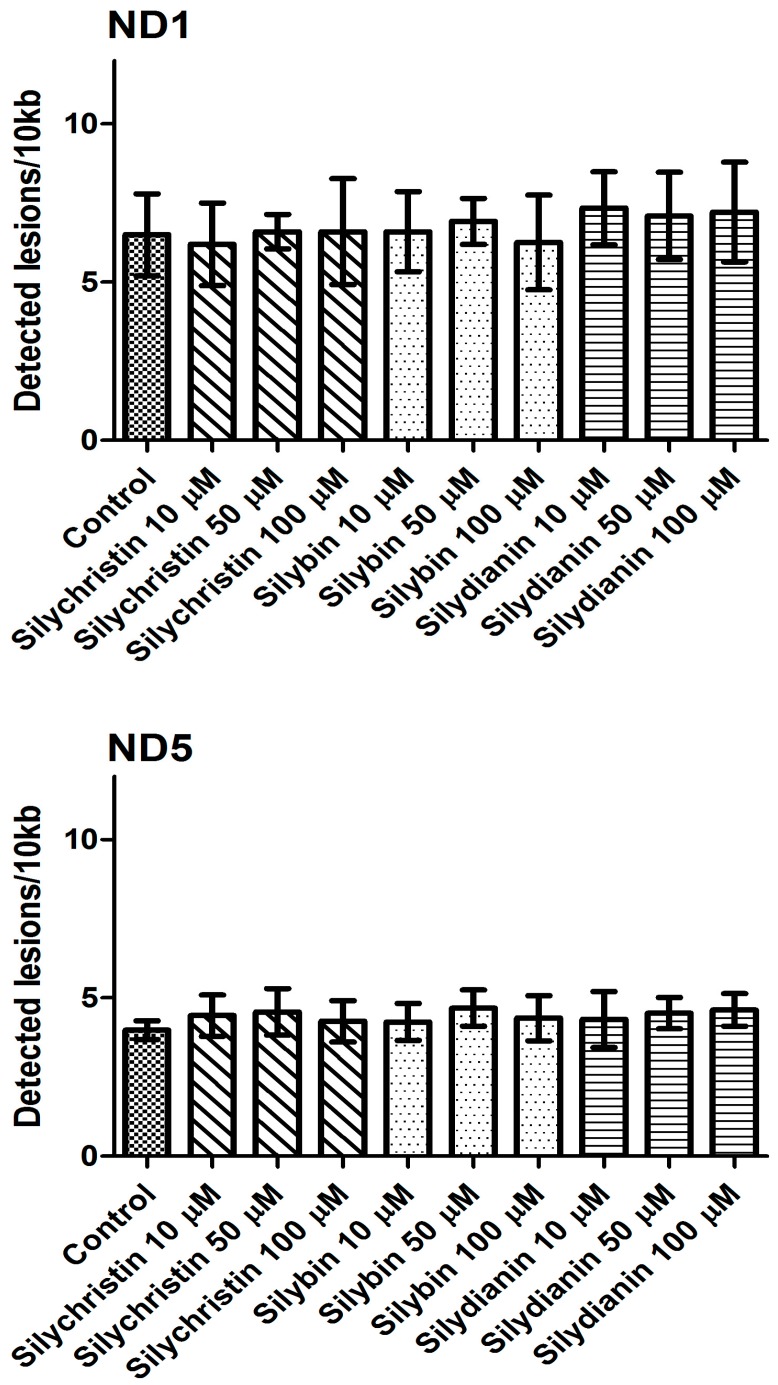
The effect of flavonolignans (silychristin, silybin and silydianin in concentrations of 10, 50 and 100 µM) on mitochondrial DNA (mtDNA) lesion frequency per 10 kb DNA in *ND1* and *ND5* genes, estimated by SLR-qRT-PCR amplification of total DNA from A549. The data represent means of ± SD, *n* = 8.

**Figure 8:**
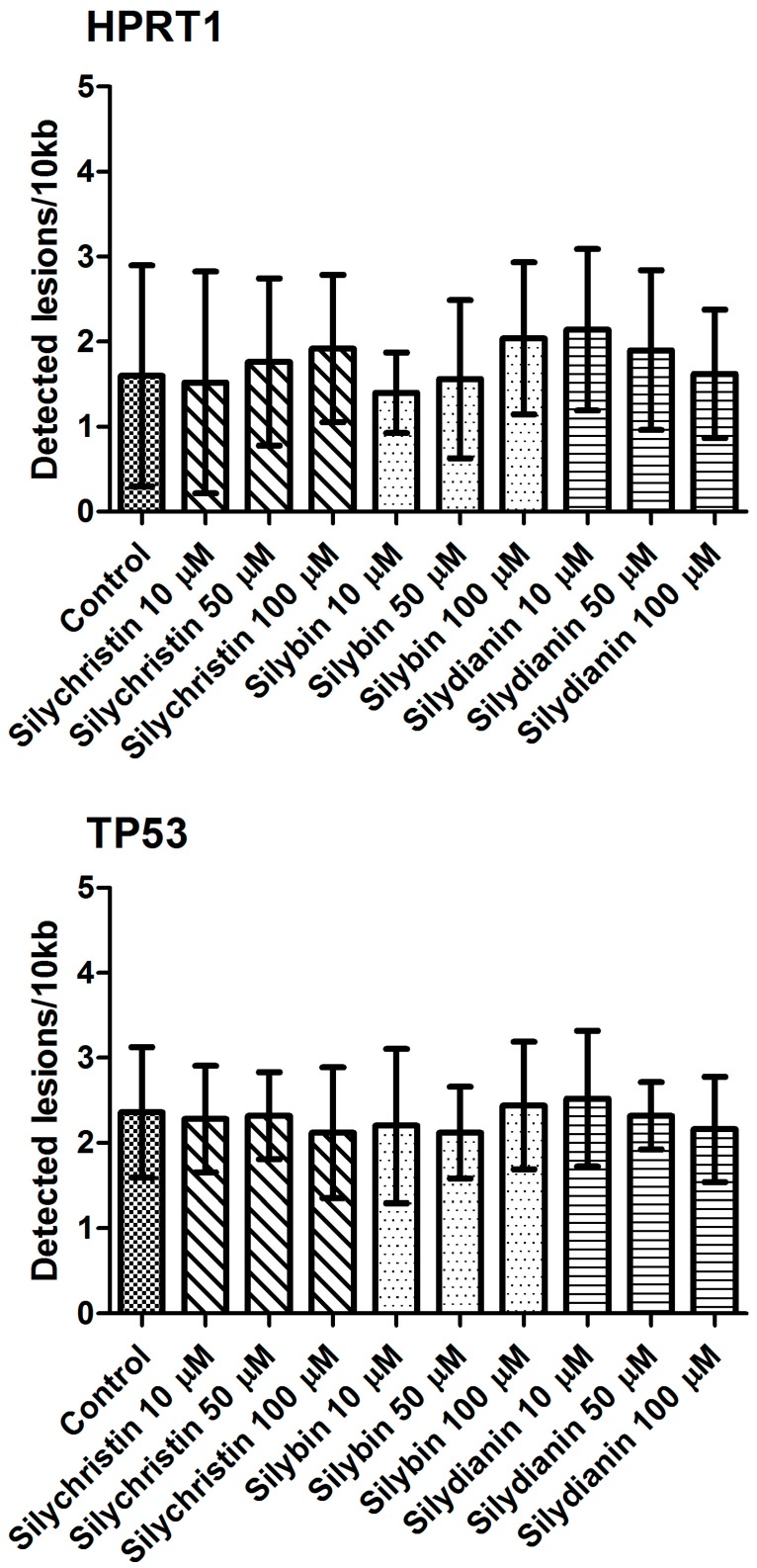
The effect of flavonolignans (silychristin, silybin and silydianin in concentrations of 10, 50 and 100 µM) on nuclear DNA (nDNA) lesion frequency per 10 kb DNA in *HPRT1* and *TP53* genes estimated by SLR-qRT-PCR amplification of total DNA from A549. The data represent means of ± SD, *n* = 8.

The next part of our study was to evaluate the genotoxicity of flavonolignans by determination of mtDNA and nuclear DNA damages. We examined the mtDNA damage by SLR-qRT-PCR amplification of DNA isolated from cells exposed to the flavonolignans: silychristin, silybin and silydianin in concentrations of 10, 50 and 100 µM for 24 h. In cell samples treated with silychristin and silybin in concentrations of 50 and 100 µM, we found a decreased level of mtDNA damage in both the *ND1* and *ND5* regions, while any differences were observed in the level of mtDNA damage between silydianin-treated samples and control samples (Figure 7). However, we did not find any differences in the level of nDNA damage in either the *HPRT1* or *TP53* genes between all samples treated with flavonolignans and the A549 control cells (Figure 8).

### 3.7. Effect of Flavonolignans on Apoptotic Genes Expression in Human Lung Cancer Cell Line A549

The results presented in Table 1 have shown that none of the tested flavonolignans have an influence on expression of the main apoptotic genes (*CASP3*, *CASP8*, *CASP9*, *BCL-2*, *BAX*, *APAF1*) under any of the tested concentrations.

## 4. Discussion

Blood platelets are the smallest un-nucleated blood cells that play a significant role in maintaining hemostasis. The average life span of the blood platelets is from 7 to 10 days, after which they are removed from the bloodstream through the reticulo-endothelial system in the spleen, liver and bone marrow [42,43]. During platelet activation O_2_⁻• and other ROS are produced, and are involved in regulation of platelet activities [44]. The O_2_⁻• is central to ROS chemistry, because it can be converted into other physiologically relevant ROS by enzymatic or non-enzymatic reactions. ROS production in activated platelets is dependent on enzymatic cascade of arachidonic acid metabolized via cyclooxygenase (COX) or 12-lipoxygenase, the glutathione (GSH) cycle and metabolism of phosphoinositides [45,46]. ROS are generated in platelets mostly by activation of NAD(P)H oxidase [47] and xanthine oxidase [48].

Blood platelets are very sensitive cells that undergo spontaneous activation after collection from the blood flow. For this reason, these materials need to be very quickly analyzed. Blood platelets after collection without any agonist generate ROS and undergo apoptosis within a few hours. In our analysis, we demonstrated not only that the tested flavonolignans are not toxic to blood platelets, but may also protect the cells against apoptosis and ROS generation. We showed reduction of O_2_⁻• generation by platelets treated with all tested flavonolignans (Figure 3B). As part of our current research, we performed studies with DCFH-DA dye. The DCFH-DA enters through the cell membrane and is enzymatically hydrolyzed with intracellular esterases to non-fluorescent DCFH, which is then oxidized to highly fluorescent dichlorofluorescin (DCF) in the presence of intracellular ROS. That analysis also shows that tested compounds reduce the intracellular ROS level in blood platelets (Figure 3A).

The studies performed by our research team in recent years [26,27,28] have shown that flavonolignans, mainly silychristin and silybin, have strong anti-platelet properties, observable as a reduction of blood platelet activation to physiological agonists such as ADP and collagen, as well as a reduction of the arachidonic acid pathway. In the current study, we documented these effects as being unrelated to flavonolignan toxicity in platelets. Additionally, we demonstrated polarization of the mitochondrial membrane of silychristin- and silybin-enhanced platelets (Figure 1). The mitochondrial membrane potential (ΔΨm) generated by proton pumps is an essential component in the process of energy storage during oxidative phosphorylation. The decrease of ΔΨm may be signal of loss of cell viability and be a cause of various pathologies [49]. Mitochondria are the primary cellular consumers of oxygen and contain numerous redox enzymes capable of transferring single electrons to oxygen, generating the O_2_⁻•. Furthermore, mitochondrial insults, including oxidative damage itself, can cause an imbalance between ROS production and removal, resulting in net ROS production [50]. It has previously been proven that silymarin has a protective effect on mitochondrial structure and function. Rolo et al. [51] showed that silybin supplementation optimizes the mitochondria electron-transport chain, decreasing electron leakage and ROS formation and directly reducing the activities of ROS-producing enzymes. The results obtained in our current study also suggest a protective effect of silychristin and silybin on spontaneous platelet mitochondrial damage, and on stabilization of the mitochondrial membrane.

There are many studies presenting flavonolignans as having strong anti-inflammatory properties [52,53,54], resulting in significantly reduced production of pro-inflammatory cytokines. In our previous study, we also documented the anti-inflammatory properties of flavonolignans on IL-1β-induced human blood samples [29]. For this reason, we decided to verify the toxic effect of flavonolignans on blood platelets’ peripheral blood mononuclear cell. The results obtained in this study confirm that the observed anti-inflammatory properties of flavonolignans are not associated with its cytotoxicity against white blood cells. We showed that these compounds have no influence on the viability of PMBCs (Figure 4).

There are also many studies that demonstrate that in selected cell lines, flavonolignans cause cell cycle arrest (G1 and G2-M arrest) and can induce apoptosis. These effects are exerted mainly through the inhibition of growth factor receptor-mediated mitogenic and cell survival signaling, particularly in the activation of tyrosine kinases [24,55]. Li et al. [56] showed that silybin induces apoptotic cell death in human malignant melanoma A375-S2 cells by increasing the expression of Fas-associated proteins with a death domain (FADD)—a downstream molecule of the death receptor pathway followed by cleavage of procaspase-8 that then induces apoptosis. It has also been demonstrated that silymarin inhibits the growth and survival of human umbilical vein endothelial cells (HUVECs), by inhibiting capillary tube formation, and induces cell cycle arrest and apoptosis while reducing invasion and migration [55]. In all our studies, the aim of which was to demonstrate flavonolignans’ health benefits, we used active concentrations (10–50–100 µM) of these compounds. They can be reached in plasma after oral administration of a novel form of Milk thistle extract, delivered in a phytosome-based, self-emulsifying and self-microemulsifying drug delivery system, as well as in the form of nano-emulsions [57,58,59]. As such, we investigated the cytotoxic and genotoxic effect of flavonolignans in 3 concentrations (10–50–100 µM) on the human lung cancer cell line A549 cell line according to standard protocol. At first, we checked the effect of flavonolignans on cell viability and morphology. We did not observe any changes between control cells and cells treated by flavonolignans. Similar results were observed for examination of nDNA damage by SLR-qRT-PCR amplification of DNA isolated from the A549 exposed to silychristin, silybin and silydianin.

Next, we investigated the effect of flavonolignans on mitochondrial DNA. First, we estimated the number of copies of mitochondrial DNA in the cells. The mtDNA copy number is a critical component of overall mitochondrial health. The mtDNA replication is carried out independently of the cell cycle by the nuclear DNA (nDNA) encoded polymerase γ, the only DNA polymerase found in the mitochondria [60]. Replication of the mtDNA is important for ensuring that cells have a sufficient number of mtDNA copies to meet their specific requirements for the generation of cellular energy through oxidative phosphorylation [61]. The results show that silybin and silychristin increased the number of mtDNA copies, while silydianin had no effect. The oxidative production of ATP required for cellular function also generates reactive oxygen species that damage mitochondrial DNA, and this ultimately results in mitochondrial dysfunction [62]. In the current study, we showed that silybin and silychristin not only have no genotoxic effect on mtDNA, but also decrease the number of mtDNA lesions.

The last parameter evaluated in this study was the expression of genes for proteins involved in apoptosis. Caspases have a proteolytic activity and are able to cleave proteins at aspartic acid residues, although different caspases have different specificities involving recognition of neighboring amino acids. Once caspases are initially activated, there seems to be an irreversible commitment towards cell death. For our analysis we selected two “initiators” (caspase 8 and 9), and one “executioner” (caspase 3). The BCL-2 proteins family is at the center of the apoptotic cascade, and determines cytochrome C release from the mitochondria, via alteration of mitochondrial membrane permeability. These proteins have special significance since they can determine whether the cell commits to apoptosis or aborts the process [63]. For our analysis, we selected one anti-apoptotic protein—Bcl-2, and one pro-apoptotic protein—Bax. Additionally, we chose to analyze APAF-1, which is an evolutionarily conserved component of the apoptosome responsible for activation of caspase 9 in the initiation of apoptosis [64]. However, we did not observe any changes in the tested genes’ expression at the mRNA level, between all samples treated with flavonolignans and the control sample of A549 cells.

For the first time in any research, we have presented in our current study’s findings that the three major flavonolignans, silybin, silychristin and silydianin, do not have any cytotoxicity and genotoxicity effects in concentrations of up to 100 µM in various cellular models. This proves that the antiplatelet and anti-inflammatory effect of these compounds is related to the molecular health mechanism. Additionally, we demonstrated that silybin and silychristin have very strong positive effects on cell mitochondria, reducing ROS formation as well as protecting them from spontaneous mtDNA damage.

After oral administration, flavonolignans undergoes extensive enterohepatic circulation which eliminated them from organism. About 80% of flavonolignans is excreted as glucuronide and sulfate conjugates with bile while 8% is excreted in an unchanged form in the urine. However the elimination half-life of flavonolingnans (based on silybin) is approximately 6 h [65], which is enough to time to act as bioactive components. Additionally, oral supplementation of novel forms of silymarin increase bioavailability of flavonolignans to concentrations used in this study. In study performed by Hwang et al. [59], the oral supplementation (equivalent to 140 mg/kg dose of silymarin) of a novel form of silymarin—silymarin/PVP/Tween 80 at a weight ratio of 5/2.5/2.5 result the maximum plasma concentration of silybin at level 44.85 ± 11.42 μg/mL, which correspond to 100 μM (maximum concentration used in this study).

## 5. Conclusions

Results presented in this study clearly suggests that tested flavonolignans can be considered to be promising nutraceuticals, not only for liver treatment, but also in cardiovascular and inflammatory disorders. Additionally, these compounds have beneficial effects on cell mitochondria.

## Figures and Tables

**Table 1:** The effect of flavonolignans (silychristin, silybin and silydianin in concentrations of 10, 50 and 100 µM) on pro-apoptotic genes’ expression in the A549 cell line. The data represent means of ± SD, *n* = 8.

Gene Expression (2^−ΔCt^)	Control	Silychristin (µM)			Silybin (µM)			Silydianin(µM)		
		10	50	100	10	50	100	10	50	100
*CASP3*	5.2 × 10^−6^ ± 6.5 × 10^−7^	5.3 × 10^−6^ ± 1.7 × 10^−7^	4.5 × 10^−6^ ± 8.5 × 10^−7^	6.1 × 10^−6^ ± 8.2 × 10^−7^	5.4 × 10^−6^ ± 8.9 × 10^−7^	5.5 × 10^−6^ ± 4.9 × 10^−7^	5.6 × 10^−6^ ± 5.7 × 10^−7^	6.2 × 10^−6^ ± 2.2 × 10^−6^	5.2 × 10^−6^ ± 4.5 × 10^−7^	5.7 × 10^−6^ ± 1.1 × 10^−7^
*CASP8*	3.8 × 10^−6^ ± 7.2 × 10^−7^	3.4 × 10^−6^ ± 6.3 × 10^−7^	3.3 × 10^−6^ ± 3.9 × 10^−7^	3.4 × 10^−6^ ± 4.2 × 10^−7^	3.2 × 10^−6^ ± 4.9 × 10^−7^	3.7 × 10^−6^ ± 8.1 × 10^−7^	3.5 × 10^−6^ ± 5.7 × 10^−7^	5.7 × 10^−6^ ± 1.2 × 10^−6^	4.6 × 10^−6^ ± 7.5 × 10^−7^	3.7 × 10^−6^ ± 2.9 × 10^−7^
*CASP9*	5.7 × 10^−6^ ± 6.9 × 10^−7^	5.5 × 10^−6^ ± 6.9 × 10^−7^	5.4 × 10^−6^ ± 1.4 × 10^−7^	5.9 × 10^−6^ ± 6.9 × 10^−7^	5.9 × 10^−6^ ± 9.9 × 10^−7^	5.7 × 10^−6^ ± 9.1 × 10^−7^	5.4 × 10^−6^ ± 9.7 × 10^−7^	5.9 × 10^−6^ ± 2.1 × 10^−7^	5.4 × 10^−6^ ± 6.4 × 10^−7^	5.3 × 10^−6^ ± 6.7 × 10^−7^
*BCL2*	5.8 × 10^−8^ ± 4.9 × 10^−9^	5.9 × 10^−8^ ± 8.4 × 10^−9^	5.3 × 10^−8^ ± 7.7 × 10^−9^	5.6 × 10^−8^ ± 5.9 × 10^−9^	4.9 × 10^−8^ ± 1.1 × 10^−8^	6.7 × 10^−8^ ± 1.0 × 10^−8^	5.8 × 10^−8^ ± 5.5 × 10^−9^	5.7 × 10^−8^ ± 8.9 × 10^−9^	6.3 × 10^−8^ ± 1.1 × 10^−8^	6.1 × 10^−8^ ± 5.7 × 10^−9^
*BAX*	5.6 × 10^−5^ ± 3.1 × 10^−6^	5.2 × 10^−5^ ± 4.3 × 10^−6^	5.5 × 10^−5^ ± 1.7 × 10^−6^	5.4 × 10^−5^ ± 3.4 × 10^−6^	5.4 × 10^−5^ ± 9.8 × 10^−6^	5.5 × 10^−5^ ± 1.1 × 10^−6^	5.5 × 10^−5^ ± 8.0 × 10^−6^	5.9 × 10^−5^ ± 9.1 × 10^−6^	5.6 × 10^−5^ ± 5.7 × 10^−6^	5.7 × 10^−5^ ± 3.3 × 10^−6^
*APAF*	3.5 × 10^−6^ ± 9.6 × 10^−7^	3.4 × 10^−6^ ± 2.3 × 10^−7^	3.8 × 10^−6^ ± 8.9 × 10^−7^	3.4 × 10^−6^ ± 2.3 × 10^−7^	3.9 × 10^−6^ ± 7.8 × 10^−7^	3.7 × 10^−6^ ± 5.9 × 10^−7^	3.6 × 10^−6^ ± 8.7 × 10^−7^	3.3 × 10^−6^ ± 4.5 × 10^−6^	3.9 × 10^−6^ ± 6.0 × 10^−7^	3.5 × 10^−6^ ± 5.7 × 10^−7^

## Supplementary Materials

The following are available online at www.mdpi.com/2072-6643/9/12/1356/s1, Table S1: Description of semi-long run RT-PCR primers used for mitochondrial and nuclear DNA damage quantification. All primers were designed with the help of the Primer3 software (http://bioinfo.ut.ee/primer3-0.4.0/) and synthesized by Sigma-Aldrich (St. Louis, MO, USA). Complete nucleotide sequences for each gene were taken from the ENSEMBL database (https://ensembl.org/). Figure S1: The absence of morphological changes in the A549 cells cultures in the presence of flavonolignans ((A) silychristin, (B) silybin, (C) silydianin) in a concentration of 100 µM. The figure here presents samples of photos obtained during the viability analysis in the cell counter.
