# Supplementary material for: Evaluation of the Cytotoxicity and Genotoxicity of Flavonolignans in Different Cellular Models
Source: Nutrients. 2017 Dec 14;9(12):1356. doi: 10.3390/nu9121356 (PMC5748806; doi:10.3390/nu9121356)
Supplement: Supplementary file 1 [file nutrients-09-01356-s001.pdf]

Table S1. Description of semi-long run RT-PCR primers used for mitochondrial and nuclear DNA damage quantification. All primers were designed with the help of the Primer3 software (http://bioinfo.ut.ee/primer3-0.4.0/) and synthesized by Sigma-Aldrich (St. Louis, Missouri, USA). Complete nucleotide sequences for each gene were taken from the ENSEMBL database (https://ensembl.org/).

Genome	Target gene	Forward primer sequences (5'→3')	Reverse primer sequence (5'→3')	Amplicon length (bp)
Mitochondrial	*ND1*(mitochondrially encoded NADH: ubiquinone oxidoreductase core subunit 1)	Long fragment: ATGGCCAACCTCCTACTCCT	Long fragment: GATGAGTGTGCCTGCAAAGA	1214
		Small fragment: CCTAAAACCCGCCACATCTA	Small fragment: GCCTAGGTTGAGGTTGACCA	124
	ND5 (mitochondrially encoded NADH: ubiquinone oxidoreductase core subunit 5)	Long fragment: TCCAACTCATGAGACCCACA	Long fragment: AGGTGATGATGGAGGTGGAG	1156
		Small fragment: AGGCGCTATCACCACTCTGT	Small fragment: TTGGTTGATGCCGATTGTAA	124
Nuclear	*TP53* (tumour protein p53)	Long fragment: GGGTGTAGATGATGGGGATG	Long fragment: AACTGCGGAATGAAACAACC	1172
		Small fragment: AAGCTGCTAAGGTCCCACAA	Small fragment: GGAAAGATCGCTCCAGGAA	56
	HPRT1 (hypoxanthine phosphoribosyltransferase 1)	Long fragment: AGGGCAAAGGATGTGTTACG	Long fragment: AGTGGTTTCTGGTGCGACTT	1018
		Small fragment: TGCTGACCTGCTGGATTACA	Small fragment: TCTACAGTCATAGGAATGGATCTATCA	69

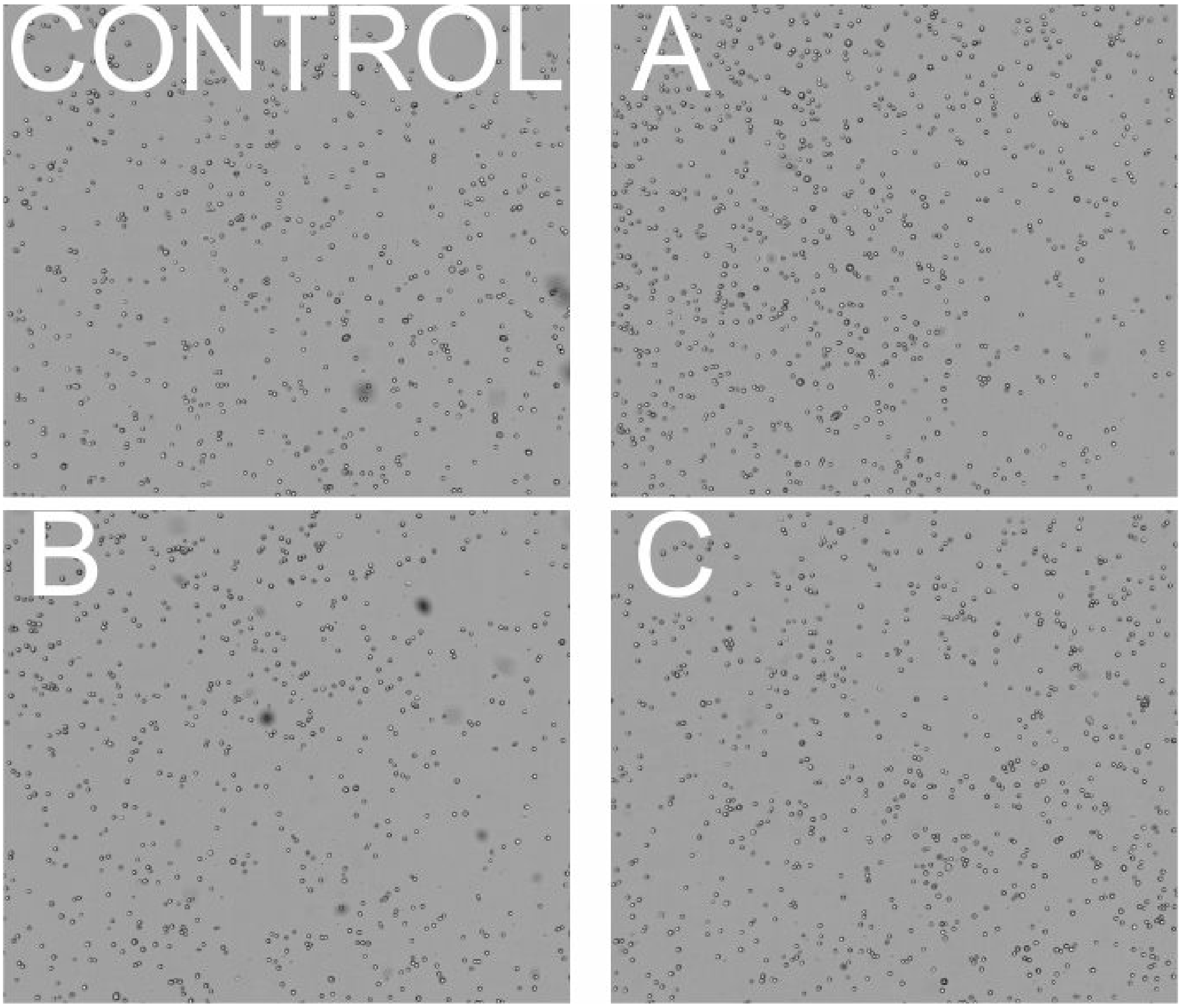

Figure S1. The absence of morphological changes in the A549 cells cultures in the presence of flavonolignans ((A) silychristin, (B) silybin, (C) silydianin) in a concentration of 100 μM. The figure here presents samples of photos obtained during the viability analysis in the cell counter.
